# Product feature and lifespan as a quality indicator for inducing eco-friendly furniture purchase

**DOI:** 10.1016/j.heliyon.2025.e42422

**Published:** 2025-01-31

**Authors:** Md. Mahiuddin Sabbir

**Affiliations:** Department of Marketing, Faculty of Business Studies, University of Barishal, Barishal-8254, Bangladesh

**Keywords:** Wood products, Engineered wood furniture, Perceived quality, Environmental concern, Subjective norms, Higher-order construct

## Abstract

Limited understanding exists of the underlying mechanisms triggering consumers' decisions to purchase Engineered Wood Furniture (EWF), particularly regarding product quality (PQ), which has typically been viewed as a unidimensional construct, neglecting its multidimensional nature. This study, therefore, captures the dimensions of PQ and identifies how environmental concerns and subjective norms interact with PQ to stimulate consumers' adoption of EWF. Analyzing data from 390 respondents, the study confirms that product features and lifespan are crucial components of PQ, directly and indirectly impacting positive attitudes and purchase intentions toward EWF. Moreover, the moderation and moderated mediation results manifested the criticality of subjective norms and environmental concerns in eliciting greater favorable attitudes and purchase intentions toward EWF based on PQ. Drawing on these theoretical contributions, the study suggests leveraging the environmental benefits of EWF in promotional activities and leveraging subjective norms through social endorsements and online communities to encourage consumers’ adoption of EWF.

## Highlights

• Product features and lifespan are crucial components of PQ.
• PQ directly impacts positive attitudes and purchase intentions toward EWF.
• PQ interacts with EC and SN to stimulate consumers' adoption of EWF.
• Marketers may leverage the environmental benefits of EWF in promotional activities.

## 1 Introduction

The rising environmental concern of consumers has led to the emergence of ‘environmental consumerism’ [1], which can be an important consideration for the furniture industry as the raw materials of solid wood furniture are primarily sourced from forests. Recognizing this, the furniture industry can potentially optimize the utilization of forest resources by not only utilizing solid wood materials but also incorporating co-products such as sawmill byproducts and wood-based panels like particleboards. Besides, as concerns about resource scarcity and energy become more pronounced, there is a growing need for innovative strategies to ensure the efficient use of raw materials [2]. Thus, eco-innovative product manufacturing has gotten much attention [[2], [3], [4]].

As public awareness regarding environmental issues continues to rise, furniture manufacturers and retailers face increasing scrutiny over their sourcing practices, with a growing emphasis on preventing deforestation [5,6]. In this context, furniture made of engineered wood products (EWPs) (e.g., plywood, laminated boards) or engineered wood furniture (EWF) could be a viable solution to address such public concern since EWPs adequately confirm both performance and sustainability issues [7,8]. Moreover, EWPs are recognized as eco-innovations that enable differentiation strategies and are linked to competitive advantages. They offer diverse environmental benefits and cost-savings, as they require fewer resources during production [2,8,9].

It would appear that the market success of eco-innovations primarily depends on consumer engagement in eco-friendly buying behavior as a means of minimizing environmental degradation and confirming the responsible allocation of resources [2,10]. However, it is concerning that the market share of eco-friendly products remains lower than recommended by idealists [[10], [11], [12]]. Since a favorable attitude toward green behavior does not inevitably lead to green behavior, an empirical examination of consumers’ purchase intention for eco-innovations (e.g., EWF) is indispensable [13]. Nonetheless, such examinations within the domain of eco-friendly furniture (e.g., EWF) are currently limited [14].

The global furniture market is projected to generate a revenue of US$766.20 billion in 2024. With a Compound Annual Growth Rate (CAGR) of 5.02 % from 2024 to 2028, this market is anticipated to grow steadily [15]. Moreover, the global engineered wood market reached a value of US$ 276.84 billion in 2021 and is projected to reach US$ 458.86 billion by the end of 2030, growing at a CAGR of 6.52 % during 2022–2030 [16]. While Asia-Pacific (e.g., India, Australia, China, Bangladesh) is anticipated to emerge as a leading market for EWPs, the penetration rate of EWPs like EWF in these regions is lower than expected and worth exploring [16]. Indeed, accelerating this penetration rate requires a comprehensive understanding of the underlying factors that influence consumers’ decisions to purchase furniture made of EWPs. However, there is a lack of studies addressing this issue, underscoring the need for further in-depth investigation into consumer behavior in this regard.

Additionally, while EWPs are used for both structural and non-structural applications, little attention has been drawn to the usage of EWPs for non-structural purposes (e.g., furniture) [17]. Besides, most existing investigations on eco-friendly furniture are primarily set in the United States [18] and Europe [19,20]. This justifies further empirical validation of existing findings or the revelation of new findings in other regions and countries worldwide [20].

In the context of durable goods like EWF purchases, product quality (PQ) is a key consideration [21,22]. However, existing literature in the furniture domain [14,20,23] often overlooked the criticality of PQ in understanding consumer behavior. Furthermore, PQ has typically been conceptualized as a unidimensional construct [22,[24], [25], [26]], neglecting its multidimensional nature, which could offer a more comprehensive understanding of consumer preferences. Furthermore, former studies have tended to view PQ solely through the lens of product quality cues and attributes (e.g., aesthetics and durability) [21] and have primarily used it as a predictor of conative responses (e.g., purchase intention) [22,24,27]. This simplistic approach fails to recognize that PQ is essentially influenced by various consumer-level psychological (e.g., environmental concern) and situational (e.g., social pressure) factors [21]. Hence, a recent systematic literature review [21] strongly advocates linking PQ with consumers’ psychological and situational factors to gain deeper insights into consumer behavior.

To address the mentioned research gaps, this study captures dimensions of PQ and identifies how consumer-level psychological (e.g., environmental concerns) and situational (e.g., subjective norms) factors interplay with PQ to stimulate consumers' attitudes and purchase intention toward EWF. Consequently, our study contributes to the existing green consumption and furniture literature by (1) assessing dimensions of PQ within the context of EWF purchase (2) evaluating the mechanisms (i.e., environmental concern and subjective norms); through which a cognitive dimension (e.g., PQ) influence consumers' affective (i.e., attitude toward EWF) and conative (i.e., purchase intention) responses. In practice, the findings of our study will offer marketers a solid understanding of the factors influencing consumers’ adoption of EWF. This insight will enable marketers to develop tailored marketing strategies that effectively target consumer preferences and motivations in the green furniture market.

## 2 Literature review and hypotheses

### 2.1 EWF

There has been mounting pressure at various local and national levels to achieve a balance in the utilization of forest resources, aiming to maximize social, economic, cultural, and environmental benefits [6]. In response, wood-polymer composites, plywood, medium-density fiberboards, and laminated boards are a group of eco-innovative materials collectively known as EWPs, contributing toward efficient resource utilization [28]. Primarily, “EWPs are made up of wood particles or fibers bound together with added resins into products that serve a variety of purposes, including structural applications and furniture” [29, p. 313]. While structural applications include using EWPs in constructing residential and non-residential multistoried buildings, EWPs are frequently used as raw materials for home and office furniture [30], commonly referred to as EWF. Thus, this study defines and examines EWF as home and/or office furniture items composed of EWPs, which are composite materials usually formed by using adhesives to join wood veneers, fibers, strands, or particles to create a stable and durable material.

Although synthetic adhesives used in EWPs are non-biodegradable, advancements in adhesive technology are addressing this limitation. Recent innovations in bio-based adhesives, such as those derived from lignin, soy protein, and other natural polymers, are reducing reliance on synthetic resins, making engineered wood more biodegradable and environmentally friendly [31,32]. Furthermore, engineered wood's overall environmental benefits, such as lower embodied energy compared to alternatives like metal or plastic and its ability to sequester carbon during its lifespan, significantly outweigh the limitations posed by adhesives [33]. Moreover, traditional furniture manufacturing often generates significant wood waste, but the production of EWPs uses smaller and otherwise discarded wood pieces, thereby reducing waste. Additionally, using wood waste and byproducts from other industries, EWF supports the closed-loop material flow emphasized in the circular economy, minimizing raw material (solid wood) extraction. This is a particularly crucial consideration for consumers' environmental sensitivity in a developing country like Bangladesh, where forest coverage is alarmingly below the recommended 25 %.

### 2.2 PQ and its dimensions

PQ refers to an individual's judgment regarding the overall excellence or supremacy of a particular product in comparison to its alternatives [34]. Garvin's [35] seminal work theorized PQ as comprising eight dimensions: performance, features, reliability, conformance, durability, serviceability, aesthetics, and perceived quality. These dimensions have been widely adopted by subsequent studies [27,[36], [37], [38]] across various industries to assess product quality. Despite a few studies [36,39] acknowledging the multidimensionality of PQ, this aspect remains largely unexplored in the existing literature, particularly within the furniture context. Understanding the multidimensionality of PQ is critical in terms of durable goods, as a product may excel in one dimension of quality but fall short in another [36]. Drawing on this research gap, Hazen [36] developed and validated four dimensions of PQ, namely features, lifespan, performance, and serviceability in a remanufactured product context. Building on their work and the extant literature [27,38,40,41], this study considered product features and lifespan as dimensions of the PQ of EWF. However, performance and serviceability were not considered, given that these dimensions typically pertain to the objective assessment of product usage, whereas this study focuses on the pre-purchase assessment of PQ for EWF.

*Product feature*: Product features are those characteristics of a product that allow users to use, apply, and possess the product to fulfill their needs and desires [42]. Product features relating to PQ can substantially influence consumers' intention to adopt a new product [36,43]. Information on product features reduces uncertainty about PQ [40]. Well-featured EWF can offer aesthetic qualities comparable to solid wood furniture. Consumers essentially value furniture that enhances the visual appeal of their homes and considers functionality and ergonomics to meet their preferences. Therefore, EWF's attractive product design and innovative features like smart storage solutions and comfortable seating arrangements can captivate consumers' attention and contribute to their perceptions of PQ. Hence, the product feature is considered a salient dimension of PQ.

*Product lifespan*: Product lifespan refers to the duration of usage prior to a product failing or degrading to the point that it becomes obsolete [36]. Scholars [38,41] contended that product lifespan may signal the quality of durable goods. Indeed, high-quality EWF is expected to withstand wear and tear over an extended period, resulting in a longer lifespan. Besides, higher-quality materials, such as high-density fiberboards or plywood, are less prone to damage or deterioration, contributing to a longer product lifespan [8] and PQ. Consequently, product lifespan is assumed to be a key dimension of PQ.

Drawing on the preceding discussions, PQ is conceptualized as a higher-order construct comprising two integral dimensions: product feature and product lifespan.

### 2.3 PQ and attitudes toward EWF

Attitude is the extent to which an individual positively or negatively assesses a certain behavior or situation [44]. Attitude is a multidimensional component comprised of cognitive, affective, and behavioral responses [45]. Based on this thought, Testa [46] examined cognitive (e.g., health belief) factors as predictors of attitude toward purchasing organic food. This is also evidenced in the furniture context, as recent studies [14,23] reported that environmental consciousness and health concerns are positively significant in triggering attitudes toward buying green furniture. Similarly, this study investigates whether PQ, a cognitive antecedent, substantially predicts consumers’ attitudes toward EWF. Relating to this issue, Munten and Vanhamme [38] found that product durability positively influences attitudes toward brands.

In fact, consumers value furniture that offers comfort, convenience, and usability. EWF, with ergonomic designs, thoughtful features, and user-friendly characteristics, can enhance consumers’ perceptions of functionality and contribute to a positive attitude toward the furniture. Moreover, positive perceptions of PQ instill trust and confidence in the product among consumers [25]. When consumers perceive EWF as being of high quality and sold by reputable brands, they are more likely to trust the brand and believe in the reliability and durability of the furniture. This positive association with quality is expected to contribute to favorable attitudes toward EWF. Accordingly, the following hypothesis is proposed:

H1 PQ positively enhances attitudes toward EWF.

### 2.4 Attitudes and purchase intentions toward EWF

According to the core reasoning of the Theory of Planned Behavior model [44], attitude is one of the key components in understanding consumers’ intention toward executing a certain behavior. Past studies [14,23,46,47] mostly demonstrated that the more the consumers perceive purchasing a certain product as desirable, wise, pleasant, and meaningful, the greater the intention toward purchasing that product. In the furniture context, the relationship between attitudes and purchase intention has been merely explored, and recent studies [14,23] have found this association to be insignificant, indicating the need for further investigation [14].

In this study's context, positive attitudes toward EWF could be linked with perceptions of quality, durability, and reliability. Consumers who hold favorable attitudes toward EWF are prone to believe that it offers comparable quality to traditional solid wood furniture, leading to higher intentions to purchase. Adding to that, consumers value furniture that enhances the visual appeal of their homes and reflects their personal style preferences. Positive attitudes toward the modern aesthetics and design versatility of EWF can drive intentions to purchase among consumers seeking contemporary furniture options. Building on this discussion, the following hypothesis is proposed:

H2 Attitude toward EWF positively enhances intentions to purchase EWF.

### 2.5 Mediating role of attitude toward EWF

Attitudes toward a certain object or behavior are often investigated as an intermediary between a cognitive stimulus (e.g., trust) and a conative outcome (e.g., purchase intentions). For example, Carfora [48] showed that trust indirectly affects intentions to buy natural foods via attitudes. Tandon [49] illustrated that product values might stimulate purchase intentions if favorable attitudes are present for eco-friendly products. The mediating role of attitude toward EWF is also grounded in the Stimulus-Organism-Response (S-O-R) [50] paradigm, illustrating how external stimuli (S) (e.g., PQ) affect people's internal states (O) (e.g., attitudes), which in turn activates cognitive and affective processes, to explain why people behave (R) the way they do (purchase intentions) [51]. Based on these backgrounds, this study assumes that PQ may indirectly induce intentions to purchase EWF by influencing the formation of positive attitudes toward EWF. Hence, it is proposed that:

H3 Attitudes toward EWF positively mediate the association between PQ and intentions to purchase EWF.

### 2.6 Moderating role of subjective norm

The subjective norm is defined as an individual's belief about whether important referents (e.g., friends, peers, family members) favor or condemn a certain behavior [52]. Multiple former studies [53,54] have demonstrated the positive moderating impact of subjective norms on the connection between cognitive predictors (e.g., environmental knowledge) and behavioral outcomes (e.g., purchase intention). Similarly, this study aims to test if subjective norm strengthens the influence of a specific cognitive factor (PQ) on a person's feelings (attitude) toward EWF. This examination builds on Hofstede Insights' [55] cultural dimension, indicating that individuals' attitudes and behavior in a collectivistic culture (e.g., Bangladesh) are expected to be swayed away by others' opinions. For example, an individual's newly purchased luxurious dining table (made from engineered wood) might positively shape his/her neighbors' or colleagues' attitudes toward EWF. The latter individual might be persuaded by the first person's endorsement of PQ relating to the practical and environmental benefits of EWF, or they might be motivated to maintain social status by following suit.

Besides, the family plays a central role in collectivistic societies, with familial ties and obligations influencing various aspects of individuals' lives, including consumer behavior [56]. Subjective norms transmitted through family networks, such as parental guidance or spousal influence, can shape individuals’ perceptions of quality and attitudes toward EWF, particularly in household decision-making. This study, therefore, posits that a positive association between PQ and attitude toward EWF is likely to be greater when the subjective norm is more pronounced in a collective society. Hence, the following hypothesis is formulated:

H4 Subjective norm positively moderates the relationship between PQ and attitudes toward EWF.

### 2.7 Moderating role of environmental concern

Environmental concern is defined as the extent to which individuals are conscious of environmental problems and lend support to resolve them or express intentions to find solutions [57]. The Value-Basis Theory proposes that environmental concern inculcates values among people for themselves, their family members and relatives, and for other living creatures (e.g., trees, animals) [58]. With growing global concerns about environmental sustainability, many consumers are increasingly conscious of the ecological impact of their actions. EWF could be perceived as a sustainable option, as it utilizes recycled solid wood waste, and the substances released from EWP adhesives are no more harmful than those from solid wood products [8]. Besides, bio-based adhesives, including those derived from lignin, soy protein, and other natural polymers, are increasingly utilized in the manufacturing of EWPs [31,32]. Hence, consumers with heightened environmental concerns are more likely to have purchase intentions [59,60] and positive attitudes toward eco-friendly products like EWF.

Specifically relevant to this study's context, product durability and reliability signal a product's environmental sustainability [62]. Durable products have longer lifespans, requiring less frequent replacement and contributing to lower waste generation [62]. Accordingly, the current study speculates that environmental concerns may positively reinforce the influence of PQ (encompassed by product features and lifespan) on attitudes toward EWF. That means environmentally concerned consumers are more inclined to consider the PQ of EWF favorably, leading to positive attitudes toward EWF. Accordingly, it is hypothesized that:

H5 Environmental concern positively moderates the relationship between PQ and attitudes toward EWF.

**Fig. 1:**
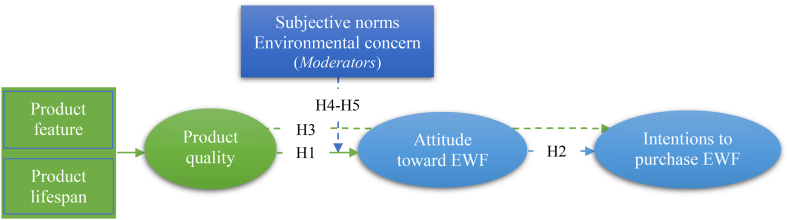
Proposed research model.

Fig. 1 displays the proposed model based on this study's hypotheses:

## 3 Method

### 3.1 Study context

The Asia-Pacific region is predicted to emerge as a leading market for EWPs [16]. Belonging to the Asia-Pacific region, Bangladesh's furniture industry is experiencing a consistent growth of 18–20 percent annually [5] owing to the rising demand for solid wood furniture and furniture made of EWPs, metal, cane, and rattan [63]. Such a growing demand for furniture makes Bangladesh (a developing country) a suitable context for this study.

### 3.2 Sample and data collection

This study used a nonprobability convenience sampling technique, where respondents were selected primarily from this interviewer's personal and social media network in Bangladesh (i.e., Facebook) [64]. Three graduates contacted potential respondents via mobile phone, email, and messenger. After explaining the study's rationale and nature, they were asked whether they were willing to participate and were familiar with EWF. A note with a survey link enclosing the study objective, request for participation, and assurance of their right to withdraw at any moment was sent to the 571 prospective respondents via messenger or email. Responses from 431 participants were received, while 390 were deemed useable after data cleaning. The G∗Power software was used to identify the sample size, which (settings: effect size = 0.15, significance criteria = 0.05, and power = 0.95) resulted in 89 as an ideal sample size for the current study. This study's sample size (n = 390) is in line with this suggested standard and comparable to former similar studies [14,23,64].

### 3.3 Measures

This study applied previously validated measures to ensure content validity [65], which were slightly modified to the current research context. A five-point Likert-type scale (1 = strongly disagree, 2 = disagree, 3 = neutral, 4 = agree, 5 = strongly agree) was employed for all measurement items except for attitude toward EWF, which was measured on a semantic differential scale (ranging from 1 to 5). These measures were further evaluated by two academic experts from marketing to avoid ambiguity and ensure precision. A list of these measures with their relevant sources is provided in Appendix A.

## 4 Analysis and results

The sample demographic characteristics were measured using SPSS and presented in Table 1. Afterward, two-stage structural equation modeling (SEM) was performed using SmartPLS 3 (bootstrapping with 5000 subsamples) [66].

**Table 1:** Sample demographics.

		Frequency	Percentage
Age (years)	18–30	160	41.0
	31–40	100	25.6
	41–50	88	22.6
	51–60	33	8.5
	More than 60	9	2.3
Household income (US$/month)	Less than 210	135	34.6
	210–419	161	41.3
	420–628	75	19.2
	629–840	13	3.3
	More than 840	6	1.5
Gender	Male	289	74.1
	Female	101	25.9
Occupation	Business owner	75	19.2
	Service holder	144	36.9
	Student	79	20.3
	Unemployed	66	16.9
	Others	26	6.7
Education	Secondary	33	8.5
	Higher secondary	60	15.4
	Bachelor's degree	140	35.9
	Master's degree	113	29.0
	Above master's degree	44	11.3

The measurement model analysis shows that factor loadings (>0.60), Cronbach's alpha (>0.70), composite reliability (>0.70), and average variance extracted (AVE) (>0.50) exceed the recommended value [66], thereby verifying the convergent validity and internal consistency of the measured items (details available in Appendix A). Regarding discriminant validity, the diagonal elements (square root of AVE) are higher than the non-diagonal elements (correlation coefficients) in the corresponding rows and columns (Table 2), satisfying the criteria for sufficient discriminant validity [67].

**Table 2:** Constructs’ correlation coefficients and square root of AVE (in bold on diagonal).

	ATT	EC	IPE	PL	PF	SN
ATT	**0.733**					
EC	0.347	**0.777**				
IPE	0.574	0.479	**0.834**			
PL	0.498	0.482	0.685	**0.800**		
PF	0.423	0.490	0.629	0.615	**0.792**	
SN	0.444	0.365	0.599	0.551	0.478	**0.847**

*Note:* ATT = attitude toward EWF; EC = environmental concerns; IPE = intentions to purchase EWF; PL = product lifespan; PF = product feature; SN = subjective norm.

A three-step technique [66] was followed to validate one higher-order formative construct. First, the convergent validity of the higher-order construct was assessed by performing a separate redundancy analysis [68] for PQ. Within the analysis, the higher-order construct is estimated in relation to an alternative single-item scale of PQ. This global single item underscores the respondents’ overall evaluation of the PQ related to EWF as a criterion construct [69]. The redundancy analysis produces path coefficients higher than 0.70 between the higher-order construct and the single-item measure of PQ, confirming the convergent validity of one higher-order construct [66,69].

Second, potential collinearity issues among the lower-order components of PQ were examined. Results (Table 3) show that VIF values are lower than the (restrictive) threshold value of 3 [70], suggesting our estimation is free from collinearity concerns.

Third, an assessment of the significance and relevance of the associations between the two first-order constructs and their corresponding higher-order construct (i.e., PQ) was performed by running bootstrapping (5000 subsamples) [66]. Such associations are termed second-order construct weights (appear as path coefficients in PLS-SEM), estimating whether conceptually developed first-order constructs significantly contribute to forming the second-order construct [66]. In Table 3, the weights are higher than 0.10, while their signs are consistent with the underlying theory [71]. Thus, these results offer adequate empirical support for PQ as formatively formed by product features and lifespan.

**Table 3:** Measurement model assessment results for formative second-order constructs (table by author).

Higher-order construct	First-order constructs	VIF Values	Outer weights	*p-value*	95 % BC CI
PQ	PF	1.608	0.538	0.000	(0.508, 0.570)
	PL	1.608	0.575	0.000	(0.542, 0.617)

*Note:* BC CI = bias-corrected confidence interval.

Drawing on the acceptable reliability and validity of the measurement model, this study undertook a structural analysis for testing H1-H3. The standardized path coefficients and their significance levels (i.e., *t-value and p-value* in Table 4) were assessed for estimating empirical validation of the structural model, suggesting the hypothesized paths (H1-H3) are significant. All other statistics, including VIF, *f*^*2*^, and Q^2^ values, are within the suggested value. The *R*^*2*^ results in Table 4 further indicate that PQ explains a 26.4 % variance in attitudes toward EWF. Moreover, attitudes toward EWF and PQ directly and indirectly explain 32.9 % variations in intentions to purchase EWF.

**Table 4:** Structural model evaluation.

Hypotheses	Structural paths	Path coefficient	*t-value*	*p-value*	95 % BC CI	VIF	R^2^	*f*^*2*^	Q^2^	Hypothesis supported?
H1	PQ→ATT	0.514	11.420	0.000	(0.413, 0.591)	1.000	0.264	0.359	0.139	Yes
H2	ATT→IPE	0.574	12.418	0.000	(0.468, 0.653)	1.000	0.329	0.491	0.225	Yes
H3	PQ→ATT →IPE	0.295	6.398	0.000	(0.201, 0.380)	1.000		0.359		Yes

*Note:* PQ = product quality; VIF = variance inflation factor; BC CI = bias-corrected confidence interval.

Moreover, one-way ANOVA was performed to examine the potential impact of respondents’ demographics on purchase intentions (see Appendix B for details). According to the results, there are no significant differences in mean purchase intentions among different household income, gender, and education groups. However, statistically significant differences in mean purchase intentions were observed across various age and occupation groups. Specifically, purchase intentions increased as the age increased. Regarding occupation, individuals engaged in business or diverse professions, such as freelancing and research and development, exhibited higher purchase intentions for EWF.

Additionally, moderation analysis was performed by SPSS PROCESS [72]. As shown in Table 5, subjective norms and environmental concerns are significant moderators in the association between PQ and attitude toward EWF. Therefore, H4 and H5 are supported, meaning the effect of PQ on fostering favorable attitudes toward EWF is stronger for consumers who are highly influenced by subjective norms and have greater environmental concerns. Moreover, the conditional indirect effects of PQ on intentions to purchase EWF through attitudes toward EWF are positive and significant [as confidence intervals (CI) do not cross 0] for lower to higher influence of subjective norms and environmental concerns (Table 6). Besides, the estimated moderated mediation values for the relationship between PQ and intentions to purchase EWF through attitudes toward EWF were β = 0.035, with CI ranging between 0.007 and 0.067 (moderator = subjective norm) and β = 0.061, with CI ranging between 0.028 and 0.109 (moderator = environmental concerns). Together, these results provide additional support to the mediating effects (H3), indicating that the indirect impact of PQ on intentions to purchase EWF through attitudes toward EWF increases if consumers are highly influenced by subjective norms and have greater environmental concerns.

**Table 5:** Moderating effects of subjective norms and environmental concerns.

Hypotheses	Interactions	Dependent variable: attitude toward EWF
H4	SN × PQ	0.122∗∗
H5	EC × PQ	0.185∗

∗p < 0.001, ∗∗p < 0.01.

**Table 6:** Conditional indirect effects for lower to higher influence of subjective norms and environmental concerns.

Paths	Level	β	Standard error	95 % CI
PQ→ATT→IPE (Moderator: SN)	Low	0.101	0.030	(0.052, 0.167)
	Medium	0.136	0.032	(0.080, 0.205)
	High	0.147	0.034	(0.086, 0.221)
PQ→ATT→IPE (Moderator: EC)	Low	0.148	0.036	(0.085, 0.226)
	Medium	0.185	0.041	(0.114, 0.271)
	High	0.221	0.047	(0.138, 0.324)

## 5 Discussions

This study elucidates the underlying mechanisms through which PQ influences consumers' intentions to purchase an eco-friendly, durable good, specifically EWF. The study's results validate product features and lifespan as pivotal constructs forming PQ, supplementing the findings of Hazen [36]. Table 4 further suggests that PQ directly enhances attitudes toward EWF (H1) and indirectly influences intentions to purchase EWF (H3), emphasizing the significant impact of PQ on consumer behavior. Complementing earlier assertions [36,45], these findings indicate that adequate features, visually appealing design, reliability, and durability of EWF would enhance consumers' intentions to purchase EWF. Accordingly, these results are potential fresh contributions to the literature, as no former study has verified such findings in an EWF context.

Additionally, results regarding H2 specify that if people consider purchasing EWF as a favorable, good, and meaningful behavior, they would be more likely to purchase EWF. Unlike previous studies in the furniture context [14,23], this study reveals a positive significant path from attitudes to purchase intentions. Moreover, this finding concurs with that of past studies in other contexts [46,73], thereby enriching the existing furniture literature by uncovering a significant affective-conative link in the unique context of EWF.

Supporting previous studies [54,61], the current results regarding H4 and H5 show that subjective norms and environmental concerns substantially moderate the direct relationship (Table 5) between PQ and attitudes toward EWF, as well as the indirect relationship (Table 6) between PQ and intentions to purchase EWF through attitudes toward EWF. These moderating impacts underscore that individuals with higher levels of environmental concerns and susceptibility to subjective norms are more likely to positively evaluate the PQ of EWF. Consequently, this positive evaluation motivates consumers to form favorable attitudes toward EWF and increases their willingness to purchase EWF. Given potential consumer skepticism about the quality of green products [25], the current findings thus suggest marketers leverage subjective norms, environmental concerns, and consumers’ attitudes to address such distrust.

Moreover, consistent with Shahsavar et al.’s [20] work, this study illustrated that consumers' demographics, particularly age and occupation, might significantly influence purchase intentions. This finding supports Shahsavar et al.’s [20] assertion that both psychographic and demographic variables can exert a substantial impact on eco-friendly furniture purchase intentions.

## 6 Theoretical contributions

This study presents three theoretical contributions to the current literature. First, drawing on Hazen's [36] work, the current study validated the multidimensionality of PQ, a less explored phenomenon, in a unique EWF context. Indeed, this work established that product feature is a crucial dimension of PQ, communicating products' aesthetics and functionality to assure consumers of their quality [40]. Besides, product lifespan is a critical aspect of PQ, signaling the reliability and durability of EWF.

Second, in response to a recent research call [21], this investigation demonstrated how consumer-level psychological (e.g., environmental concerns) and situational (e.g., subjective norms) factors interplay with PQ to stimulate consumers’ attitudes and purchase intentions toward EWF. The moderation results manifested the importance of subjective norms and environmental concerns in eliciting a greater level of favorable attitudes toward EWF based on PQ. Besides, the moderated mediation results outlined the criticality of subjective norms and environmental concerns as moderators, as well as the attitudes of EWF as a mediator in the connection between PQ and purchase intentions.

Third, by confirming the linkage from PQ to attitudes to purchase intentions, notably the significant mediation effect of attitudes, this paper substantiates the efficacy of S-O-R [50] and CAC (cognitive-affective-conative) [74] frameworks. This substantiation highlights that consumers’ decision-making regarding durable goods purchase (conative action) is not solely influenced by cognitive and external stimuli (e.g., PQ) but rather necessitates an ideal connection between cognitive stimuli and affective states.

## 7 Insights for marketers

Given the significant impact of PQ on attitudes and purchase intentions toward EWF, it is imperative to prioritize efforts aimed at enhancing durability and instilling consumer trust in EWF's quality through promotional activities. EWF producers and marketers should uphold uncompromising standards to ensure the production of the highest quality furniture. Additionally, specific attention should be directed toward enhancing product features. With the proliferation of social media platforms such as Facebook groups and fan pages, consumers are increasingly informed about the diverse product features. Therefore, EWF designers should focus on developing visually appealing designs using technologies such as AI, offering innovative features (e.g., multipurpose beds combining bed and table functionalities), and enhancing aesthetics to align with consumers' expectations. Marketers can leverage the concept of ‘value co-creation’ [75] through internet platforms to involve consumers in the design process, thereby enhancing engagement and fostering favorable attitudes toward EWF.

Drawing on the significance of environmental concerns illustrated in this study, a comprehensive promotion with TV advertisements, banners, and billboards could be launched, highlighting the positive outcomes of purchasing EWF compared to solid wood furniture. In this context, manufacturers might underline the environmental benefits of EWF (e.g., EWF uses byproducts from other industries and reduces wood waste). Given the increasing environmental consciousness among consumers [46], marketers can appeal to this segment by showcasing videos explaining how EWF serves as a sustainable alternative. These videos can highlight EWF's use of recycled solid wood waste and reassure consumers that the substances released from EWP adhesives are no more harmful than those from solid wood products [8].

Since purchase intentions tend to increase with age, marketing efforts should focus on highlighting the durability and environmental advantages of EWF to attract older consumers who value long-lasting, high-quality products. The findings also reveal that individuals in business or diverse professions exhibit higher purchase intentions than students or service holders. To engage these segments, marketers could design campaigns emphasizing professional, modern, and customizable furniture suitable for home offices or multifunctional spaces. Positioning EWF as a smart and sustainable investment is likely to resonate well with professionals and entrepreneurs. While demographics such as income, gender, and education showed no significant differences, the growing global focus on sustainability suggests that environmental messaging could be universally effective.

Moreover, people are predominantly stimulated by their peers, friends, and family members in a collective society, as evidenced by this study's finding highlighting subjective norms as a positive moderator. Hence, furniture manufacturers and marketers can reinforce the effect of subjective norms by (1) leveraging endorsements from renowned social figures such as celebrities and role models and (2) fostering online communities through social media platforms like Facebook, LinkedIn, and Instagram to reach different professional and age groups. These platforms can facilitate interactive activities, such as polls and discussions, allowing consumers to share their post-purchase experiences regarding EWF quality, particularly in terms of durability and design.

## 8 Limitations and directions for further study

This study has some limitations that warrant consideration in future research endeavors. *First*, the focus on evaluating consumers' intentions rather than buying behavior may restrict an inclusive understanding of their purchasing patterns. Since consumers' intentions do not essentially translate into behaviors, future investigators may examine if EWF purchase intention leads to actual buying behavior. In this context, future studies could explore how consumer preferences for technological advancements and regulatory changes influence attitudes, purchase intentions, and behaviors toward EWF. *Second*, the exclusive collection of responses from a developing country may limit the generalizability of the study's findings to developed nations. Conducting a comparative study across different cultural contexts could provide additional insights into the adoption of EWF. *Third*, demographic variables could be added as independent predictors or moderators by extending this study's model, given that such variables exert a substantial impact on consumers' buying decisions [20].

## Ethics and consent section

Ethical clearance for this study is waived as it exclusively investigates consumer attitudes and intentions and does not experiment with any humans or animals.

The authors secured full consent from the respondents through the questionnaire during the data collection process. Respondents were thoroughly briefed on their rights and the objectives of this study. They could withdraw from the survey at any point during the research. Consent was obtained to utilize their responses for academic research purposes.

## Data availability statement

Data will be made available on request. For requesting data, please write to the corresponding author at mmsabbir@bu.ac.bd.

## Declaration of competing interest

The author declares no potential conflict of interest.

## Figures and Tables

**Table:** 

Construct	Items	Factor loadings∗	α	CR	AVE	Source
Product feature (PF)	An adequate number of features are available for EWF	0.820	0.801	0.870	0.628	[36]
	I consider EWF to be visually appealing	0.695				
	I can choose from all the options/designs I want for EWF	0.826				
	The appearance of EWF meets expectations	0.822				
Product lifespan (PL)	EWF has an adequate lifespan	0.803	0.811	0.876	0.639	[36]
	I consider EWF to be reliable	0.842				
	I consider EWF to be a durable product	0.806				
	EWF meets standards	0.743				
Attitude toward EWF (ATT)	For me, purchasing EWF is –					
	Extremely unfavorable = 1-Extremely favorable = 5	0.724	0.856	0.890	0.537	[14]
	Extremely bad = 1-Extremely good = 5	0.756				
	Extremely undesirable = 1-Extremely desirable = 5	0.666				
	Extremely unpleasant = 1-Extremely pleasant = 5	0.750				
	Extremely unwise = 1-Extremely wise = 5	0.673				
	Extremely meaningless = 1-Extremely meaningful = 5	0.762				
	Extremely negative = 1-Extremely positive = 5	0.792				
Subjective Norm (SN)	Most people who are important to me think I should purchase EWF	0.871	0.803	0.884	0.717	[14]
	Most people who are important to me would want me to purchase EWF	0.866				
	People whose opinions I value would prefer me to purchase EWF	0.802				
Environmental concern (EC)	I am extremely worried about the state of our environment	0.755	0.836	0.884	0.603	[14]
	To achieve sustainable development, I think humans should live in harmony with nature	0.807				
	The balance of nature is very delicate and easily upset	0.745				
	I am willing to control my consumption to protect the environment	0.771				
	I think individuals have the responsibility to protect the environment	0.803				
Intentions to purchase EWF (IPE)	I am willing to purchase EWF	0.831	0.854	0.901	0.696	[14]
	I try to purchase EWF	0.851				
	I plan to purchase EWF	0.860				
	I will make an effort to purchase EWF	0.794				

Note: CR=Composite reliability; AVE = Average variance extracted; α = Cronbach's alpha.

∗All factor loadings (λ) are significant at p < 0.001.

**Table:** 

Age	N	Mean	Std. Deviation	Std. Error	95 % Confidence Interval for Mean	
					Lower Bound	Upper Bound
18–30	160	3.5188	0.86673	0.06852	3.3834	3.6541
31–40	100	3.8000	0.71598	0.07160	3.6579	3.9421
41–50	88	3.9460	0.58286	0.06213	3.8225	4.0695
51–60	33	3.9470	0.65478	0.11398	3.7148	4.1791
More than 60	9	4.3611	0.50173	0.16724	3.9754	4.7468
Total	390	3.7429	0.77276	0.03913	3.6660	3.8199

(F = 7.508, Sig = 0.000).

**Table:** 

Household income (US$/month)	N	Mean	Std. Deviation	Std. Error	95 % Confidence Interval for Mean	
					Lower Bound	Upper Bound
Less than 210	135	3.6907	0.86344	0.07431	3.5438	3.8377
210–419	161	3.7671	0.72894	0.05745	3.6536	3.8805
420–628	75	3.7400	0.66455	0.07674	3.5871	3.8929
629–840	13	3.7692	0.96535	0.26774	3.1859	4.3526
More than 840	6	4.2500	0.54772	0.22361	3.6752	4.8248
Total	390	3.7429	0.77276	0.03913	3.6660	3.8199

(F = 0 0.842, Sig = 0.499).

**Table:** 

Gender	N	Mean	Std. Deviation	Std. Error	95 % Confidence Interval for Mean	
					Lower Bound	Upper Bound
Male	289	3.7595	0.75986	0.04470	3.6715	3.8475
Female	101	3.6955	0.81056	0.08065	3.5355	3.8556
Total	390	3.7429	0.77276	0.03913	3.6660	3.8199

(F = 0.512, Sig = 0.475).

**Table:** 

Occupation	N	Mean	Std. Deviation	Std. Error	95 % Confidence Interval for Mean	
					Lower Bound	Upper Bound
Business	75	3.9467	0.60305	0.06963	3.8079	4.0854
Service holder	144	3.8247	0.71759	0.05980	3.7064	3.9429
Student	79	3.1614	0.90939	0.10231	2.9577	3.3651
Unemployed	66	3.9242	0.58666	0.07221	3.7800	4.0685
Others	26	4.0096	0.63435	0.12441	3.7534	4.2658
Total	390	3.7429	0.77276	0.03913	3.6660	3.8199

(F = 16.966, Sig = 0.000).

**Table:** 

Education	N	Mean	Std. Deviation	Std. Error	95 % Confidence Interval for Mean	
					Lower Bound	Upper Bound
Secondary	33	3.9697	0.69809	0.12152	3.7222	4.2172
Higher secondary	60	3.6083	0.79106	0.10213	3.4040	3.8127
Bachelor's degree	140	3.6768	0.83671	0.07072	3.5370	3.8166
Master's degree	113	3.7434	0.70351	0.06618	3.6122	3.8745
Above master's degree	44	3.9659	0.70627	0.10647	3.7512	4.1806
Total	390	3.7429	0.77276	0.03913	3.6660	3.8199

(F = 2.371, Sig = 0.052).
